# Linking Photophobia, Sleep Disturbances, and Migraine Chronicity: Evidence From a Retrospective Analysis

**DOI:** 10.1002/brb3.71311

**Published:** 2026-03-25

**Authors:** Seden Demirci, Ezgi Uludüz, Sevim Eyüboğlu, Fatma Yardibi, Damla Hazal Sucu, Utku Topbaş, Aynur Özge, Derya Uludüz

**Affiliations:** ^1^ Department of Neurology, School of Medicine Akdeniz University Antalya Türkiye; ^2^ Medical Faculty, Koc University Istanbul Türkiye; ^3^ Department of Psychology Brain360 Integrative Brain Health Center Istanbul Türkiye; ^4^ Faculty of Communication, Department of New Media and Communication Akdeniz University Antalya Türkiye; ^5^ Department of Biostatistics and Medical Informatics Mersin University School of Medicine Mersin Türkiye; ^6^ School of Medicine Mersin University Mersin Türkiye; ^7^ Department of Neurology Norom Neuroscience and Excellence Center Ankara Türkiy; ^8^ Medical Faculty Istanbul Cerrahpasa University Istanbul Türkiye

**Keywords:** chronic migraine, medication overuse, migraine, network analysis, photophobia, sleep disturbances

## Abstract

**Background:**

Interictal photophobia and sleep disturbances are common in migraine patients and may contribute to migraine chronicity. However, the interplay between these symptoms and their role in the progression from episodic migraine (EM) to chronic migraine (CM) is not fully understood. This study investigates the association of ictal and interictal photophobia and sleep disturbances with CM.

**Methods:**

We conducted a retrospective analysis of 500 patients diagnosed with migraine based on the International Classification of Headache Disorders (ICHD‐3) criteria. Patients were classified into EM and CM groups. Data on photophobia, sleep disturbances, and clinical characteristics were collected. Logistic regression and network analyses were performed to identify predictors and explore symptom interconnections.

**Results:**

Among 500 participants (mean age: 41.03 ± 11.06 years; 84.2% female), 311 had EM, and 189 had CM. Ictal photophobia was present in 89.8% and interictal photophobia in 93.4% of patients, with 61.0% reporting severe interictal photophobia. Sleep disturbances were common, with 44.9% reporting difficulty falling asleep. Logistic regression identified medication overuse (OR: 5.007, 95% CI: 3.01–8.31, *p* < 0.001) and aura (OR: 0.226, 95% CI: 0.07–0.71, *p* = 0.012) as significant predictors of CM. Network analysis revealed stronger associations between interictal photophobia, daytime sleepiness, and medication overuse in CM compared to EM.

**Conclusions:**

Interictal photophobia and sleep disturbances are significant factors in the chronicity of migraine. These findings suggest that managing these symptoms may reduce migraine progression. Further research is needed to explore targeted interventions for CM patients.

## Graphical abstract

This study demonstrated that ictal photophobia was present in 89.8% and interictal photophobia in 93.4% of patients. Sleep disturbances were common, with 44.9% reporting difficulty falling asleep. Network analysis revealed stronger associations between interictal photophobia, daytime sleepiness, and medication overuse in chronic migraine compared to episodic form.

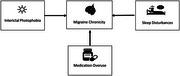

**Abbreviations**
CM: Chronic migraine
EM: Episodic migraine

## 1 Introduction

Migraine is a prevalent and disabling neurological disorder characterized by recurrent headache attacks often accompanied by associated symptoms, such as nausea, vomiting, and hypersensitivity to light and sound. It affects more than 1 billion people worldwide. The pathophysiology of migraine is complex and involves a combination of genetic, environmental, and neurovascular factors. The disorder's impact on quality of life is substantial, as migraine attacks can interfere with daily activities, work, and social engagements. It is estimated that migraine results in over 45 million disability‐adjusted life years globally, highlighting its significant contribution to the burden of disease (GBD 2016 Headache Collaborators 2018).

Photophobia is an excessive sensitivity to light and one of the basic criteria for the diagnosis of migraine. During an active migraine attack, photophobia is one of the most common and debilitating symptoms, often intensifying the pain and discomfort. Photophobia can be seen not only in the ictal period but also in the interictal period. In a large‐scale survey, over 92% of 2735 participants indicated that they used a dark room during a migraine attack, and more than 83% considered it a regular method for managing their pain (Malone et al. 2015). In a study by Mulleners et al. (2001), 99 participants tested during a migraine‐free period exhibited higher levels of photophobia and lower tolerance to visual stress from black‐and‐white contrast than 101 healthy control subjects. Higher levels of photophobia are not merely bothersome but are associated with significant work‐related disability, reduced activity levels, and impaired quality of life (Leibovit‐Reiben et al. 2024). Photophobia reflects ongoing neurovascular changes and central sensitization that extend beyond the acute phase of headache and are linked to migraine persistence and chronicity. Patients with increased sensitivity to light between migraine attacks may have a higher likelihood of experiencing new episodes, further complicating the management of the disorder (Pinheiro et al. 2021).

Sleep disturbances are a frequently reported comorbidity in individuals with migraine. The relationship between migraine and sleep is complex, with sleep disruptions potentially contributing to the onset of migraine episodes and, conversely, migraine attacks interfering with sleep quality. The neurobiological mechanism underlying this relationship is likely due to some overlapping pathophysiologies and shared anatomical structures in the brain. Sleep disorders such as insomnia, fragmented sleep, and irregular sleep patterns have been shown to exacerbate migraine symptoms. They may play a role in the progression of episodic migraine to chronic migraine (Vgontzas and Pavlović 2018).

Emerging evidence suggests that photophobia and sleep disturbances are closely interrelated in migraine patients and may interact through shared pain and circadian regulatory pathways (Sharp et al. 2024). However, the combined contribution of these symptoms to migraine chronification and their interactions at the symptom network level have not been sufficiently characterized. Addressing this knowledge gap may provide a better understanding of migraine progression mechanisms.

In this context, network analysis offers a valuable approach for addressing this complexity by enabling the visualization and quantification of relationships among multiple symptoms within an integrated framework. Unlike traditional analytical methods, network analysis allows identification of central symptoms that may play key roles in disease progression. Therefore, this study aimed to investigate whether ictal and interictal photophobia, together with sleep disturbances, are associated with migraine chronicity and may be involved in processes related to chronic migraine development.

## 2 Methods

### 2.1 Study Subjects and Design

This retrospective analysis utilized data from two outpatient headache clinics in neurology departments. A total of 500 patients with migraine, diagnosed by a neurologist according to the criteria of the third edition of the International Classification of Headache Disorders (ICHD‐3), were included. Individuals who have EM or CM, with or without aura, were included (Headache Classification Committee of the International Headache Society [IHS], 2018). Patients under 18 years of age were excluded from the study. Photophobia resulting from ophthalmological or systemic diseases was also excluded. Photophobia caused by medications other than acute migraine medications, or overuse, was also excluded.

The medical records of patients presenting with headaches were examined retrospectively. Demographic and clinical data, including the occurrence of aura, nausea, phonophobia, photophobia, osmophobia, allodynia, pain character, disease duration and severity, migraine localization, factors that could trigger migraine attacks, medication overuse, interictal photophobia, intensity of interictal photophobia, and sleep disturbances, were gathered. Photophobia severity was graded based on patient self‐reports during clinical evaluation. Patients were asked to categorize their light sensitivity as mild, moderate, or severe, and the responses were recorded in their medical records. Although this method is not based on a validated scale, it aligns with standard clinical practice in headache clinics.

Missing data were handled using a complete‐case analysis approach for primary analyses. Patients with missing information in critical variables (e.g., photophobia severity, sleep disturbances) were excluded from the corresponding analyses. Sensitivity analyses were not performed due to the low rate of missing data across key variables. Medication overuse was defined as the regular consumption of acute migraine medications for more than 3 months, including acetylsalicylic acid, nonsteroidal anti‐inflammatory drugs, and acetaminophen for at least 15 days per month, as well as ergotamines, triptans, opioids, or combination analgesics for at least 10 days per month.

Based on patient self‐reports, photophobia was classified as ictal (occurring during migraine attacks) or interictal (occurring between migraine attacks). The intensity of interictal photophobia was categorized as mild, moderate, and severe. Parameters related to sleep disturbances were identified based on patient reports of difficulty falling asleep, daytime sleepiness, waking up during the night, experiencing nightmares, and difficulty getting up. The severity of the headache was assessed using the VAS (Visual Analog Scale), ranging from 1 to 10.

The study was conducted in accordance with the principles of the Declaration of Helsinki and local laws and regulations. It received approval from the Ethics Committee of Akdeniz University Faculty of Medicine (Approval Date: April 25, 2024, Decision No: 254).

### 2.2 Statistical Analysis

The Kolmogorov–Smirnov test was performed to assess the normality of the data distribution. Descriptive statistics were used to summarize the demographic and clinical characteristics of the study population. Continuous data were presented as mean ± standard deviation (SD), while categorical data were reported as frequencies and percentages. Pearson's chi‐square test and Fisher's exact test were utilized to analyze categorical variables and compare the frequencies of occurrence. The independent‐samples *t*‐test was applied to compare normally distributed continuous variables. At the same time, the Mann–Whitney *U* test was used for variables that did not follow a normal distribution. Logistic regression models were constructed for chronic migraine. Photophobia, triggers, interictal photophobia severity, sleep‐related parameters, medication overuse, aura, and headache severity were included as predictor variables. Statistical significance was set at *p* < 0.05. Backward logistic regression was used to identify significant predictors of chronic migraine (CM) over episodic migraine (EM). This method was chosen for its ability to handle multiple variables by iteratively removing the least significant predictors to optimize model fit. This approach is particularly advantageous in exploratory analyses, where the goal is to identify key contributors to an outcome.

The network graphs were created using correlation analysis to visualize the relationships between variables. A threshold of *p* < 0.05 was used to determine the significance of correlations, which were then visualized as edges in the network graphs. In the graphs, nodes represent variables, and edges represent correlations between these variables. Positive correlations are shown in blue, while negative correlations are represented in red. The edges' thickness reflects the magnitude of the correlation coefficients, with thicker lines indicating stronger relationships. The network graphs for chronic and episodic migraine patients illustrate the correlation structure between variables. The network graph was created using JASP 0.14.1.0 software.

To assess the robustness of network structures, supplementary analyses included partial correlations adjusted for age, sex, and medication overuse (MOH), bootstrap resampling (1000 iterations) to estimate 95% confidence intervals for edge weights, and comparisons of node centrality measures between EM and CM. The EM network exhibited two small and statistically unstable negative correlations (Aura–Interictal photophobia and Aura–Trigger), which were further visualized and discussed in Figures S1–S3. The primary analyses were based on complete‐case data (main analysis). In contrast, supplementary and sensitivity analyses—such as partial correlation networks, bootstrapping, and comparisons of node centrality—were performed to assess robustness. All analyses were conducted using JASP version 0.14.1.0. Supplementary network figures were generated with the “qgraph” package (Epskamp et al. 2012) under a fixed random seed (set.seed = 1234) to ensure reproducibility.

## 3 Results

### 3.1 Demographic and Clinical Characteristics

A total of 500 participants were included in this study. 311 (62.2%) participants were diagnosed with EM, while 189 (37.8%) had CM. The mean age of the participants was 41.03 ± 11.06 years, with ages ranging from 18 to 75. Additionally, 420 individuals (84.2%) were women. The demographic and clinical data of the participants are presented in Table 1. Ictal photophobia was present in 89.8% of the migraine patients, and interictal photophobia was present in 93.4% of the migraine patients.

**TABLE 1:** The demographic and clinical characteristics of the individuals.

Characteristics	All patients (*n* = 500)
Age, mean (SD)	41.03 (11.06)
Sex, female, *n* %	420 (84.2%)
Disease duration (years), mean (SD)	18.76 (11.66)
Aura, *n* %	40 (8.0%)
Nausea, *n* %	435 (87.2%)
Vomiting, *n* %	126 (25.3%)
Photophobia, *n* %	448 (89.8%)
Phonophobia, *n* %	402 (80.6%)
Osmophobia, *n* %	184 (36.9%)
Allodynia, *n* %	33 (6.6%)
Unilateral pain, *n* %	266 (53.4%)
Pain character	
Throbbing, *n* %	429 (86.1%)
Piercing, *n* %	10 (2.0%)
Tightening	6 (1.2%)
Pressing, *n* %	8 (1.6%)
Stabbing	2 (0.4%)
Others, *n* %	43 (8.6)
Localization	
Orbita, *n* %	158 (31.7%)
Temple, *n* %	138 (27.7%)
Occipital, *n* %	85 (17.1%)
Vertex, *n* %	23 (4.6%)
Frontal, *n* %	20 (4.0%)
Generalized, *n* %	20 (4.0%)
Frontoparietal, *n* %	19 (3.8%)
Others, *n* %	35 (7)
Pain intensity (VAS), mean (SD)	8.1 (1.06)
The most trigger	
Stress, *n* %	109 (22.2%)
Hunger, *n* %	66 (13.4%)
Menstruation, *n* %	79 (16.1%)
Lack of sleep/oversleeping, *n* %	51 (10.4%)
Climate change, *n* %	21 (4.3%)
Intense physical activities, *n* %	43 (8.8%)
Alcohol	21 (4.3%)
Missing meals	32 (6.5%)
Travelling	34 (6.9%)
Odors	21 (4.3%)
Others, *n* %	14 (2.9%)
Medication overuse, *n* %	169 (33.8%)
Interictal photophobia, *n* %	409 (93.4%)
Interictal photophobia severity	
Mild, *n* %	55 (13.4%)
Moderate, *n* %	105 (25.6%)
Severe, *n* %	250 (61.0%)
Sleep disturbances	
Difficulty falling asleep, *n* %	192 (44.9%)
Daytime sleepiness, *n* %	139 (33.6%)
Nighttime awakening, *n* %	181 (43.1%)
Experiencing nightmares, *n* %	52 (12.7%)
Difficulty getting up, *n* %	190 (44.6%)

SD, standard deviation; VAS, Visual Analog Scale.

No significant differences were found between the EM and CM groups regarding age (*p* = 0.655) and sex distribution (*p* = 0.460) (Table 2). However, the duration of migraine disease was significantly longer in the CM group (20.10 ± 12.42 years) compared to the EM group (17.95 ± 11.11 years; *p* = 0.046).

- 169 patients (33.8%) had medication overuse. Among these, 88.2% had ictal photophobia, compared to 90.6% of patients without medication overuse (*p* = 0.394). Similarly, 95.5% of patients with medication overuse reported interictal photophobia, while 92.2% of those without medication overuse experienced interictal photophobia (*p* = 0.182).
- Ictal photophobia was present in 82.5% of the migraine patients with aura and in 90.4% of the patients without aura. Interictal photophobia was observed in 94.3% of migraine patients with aura and 93.3% of those without aura.

**TABLE 2:** The demographic and clinical characteristics of episodic and chronic migraine patients.

Characteristics	Episodic migraine (*n* = 311, 62.2%)	Chronic migraine (*n* = 189, 37.8%)	*p* value
Age, mean (SD)	39.99 (10.85)	42.75 (11.25)	0.655
Sex, female, *n* %	258 (83.2%)	162 (85.7%)	0.460
Disease duration (years), mean (SD)	17.95 (11.11)	20.10 (12.42)	0.046
Aura, *n* %	36 (11.6%)	4 (2.1%)	<0.001
Photophobia, *n* %	275 (88.7%)	173 (91.5%)	0.312
Pain intensity (VAS), mean (SD)	8.0 (1.09)	8.1 (1.01)	0.240
Medication overuse, *n* %	61 (19.6%)	108 (57.1%)	<0.001
Interictal photophobia, *n* %	259 (94.2%)	150 (92.0%)	0.380
Interictal photophobia severity			0.498
Mild, *n* %	31 (11.9%)	24 (16.0%)	
Moderate, *n* %	67 (25.8%)	38 (25.3%)	
Severe, *n* %	162 (62.3%)	88 (58.7%)	
Sleep disturbances			
Difficulty falling asleep, *n* %	111 (41.3%)	81 (50.9%)	0.051
Daytime sleepiness, *n* %	86 (33.0%)	53 (34.6%)	0.725
Nighttime awakening, *n* %	110 (41.4%)	71 (46.1%)	0.343
Experiencing nightmares, *n* %	34 (13.1%)	18 (11.8%)	0.705
Difficulty getting up, *n* %	111 (41.6%)	79 (49.7%)	0.103

SD, standard deviation; EM, episodic migraine; CM, chronic migraine; VAS, Visual Analog Scale.

Medication overuse was significantly higher in CM patients (57.1%) than in EM patients (19.6%; *p* < 0.001). Aura was more common in the EM group (11.6%) than in the CM group (2.1%; *p* < 0.001).

### 3.2 Photophobia Findings

Ictal photophobia was present in 89.8% of migraine patients, and interictal photophobia was present in 93.4%. Among those reporting interictal photophobia, 61.0% described it as severe, 25.6% as moderate, and 13.4% as mild. The frequency of photophobia and interictal photophobia did not differ significantly between the EM and CM groups (*p* = 0.312 and 0.380, respectively).

In the CM group, 58.7% of patients reported severe interictal photophobia, compared to 62.3% in the EM group, highlighting similar severity distributions (*p* = 0.498). While photophobia was common in both groups, interictal photophobia severity showed a high prevalence of severe forms, indicating its potential role in migraine progression.

### 3.3 Sleep Disturbances

Sleep disturbances were frequently reported among all participants. Difficulty falling asleep was the most common sleep‐related symptom, affecting 44.9% of patients, followed by nighttime awakening (43.1%) and daytime sleepiness (33.6%). Other reported symptoms included difficulty getting up (44.6%), and experiencing nightmares (12.7%).

Although sleep disturbances were more common in the CM group than in the EM group, none of the differences reached statistical significance. For instance, 50.9% of CM patients reported difficulty falling asleep compared to 41.3% of EM patients (*p* = 0.051). These findings highlight the role of sleep disturbances in both migraine subtypes, with a potential trend toward a more significant impact in CM.

### 3.4 Logistic Regression Analysis

Multivariable logistic regression analysis showed that the significant and independent predictors of CM were:

- Medication overuse (coefficient of regression: 1.611, SE: 0.259, OR: 5.007, 95% CI: 3.01–8.31, *p* < 0.001)
- Aura (coefficient of regression: –1.488, SE: 0.590, OR: 0.226, 95% CI: 0.07–0.71, *p* = 0.012)

These findings are detailed in Table 3.

**TABLE 3:** Logistic regression analysis of variables for chronic migraine.

Predictive variable	Regression coefficient	SE	OR	95% confidence interval	*p* value
Aura	−1.488	0.590	0.226	0.07–0.71	0.012
Medication overuse	1.611	0.259	5.007	3.01–8.31	<0.001
Photophobia	0.498	0.443	1.646	0.69–3.92	0.260
Pain intensity (VAS)	−0.002	0.122	0.998	0.78–1.26	0.988
Interictal photophobia	−0.553	0.481	0.575	0.22–1.47	0.250
Difficulty falling asleep	0.378	0.270	1.459	0.86–2.47	0.162
Daytime sleepiness	0.077	0.280	1.080	0.62–1.87	0.784
Nighttime awakening	−0.048	0.281	0.953	0.54–1.65	0.864
Experiencing nightmares	−0.402	0.383	0.669	0.31–1.41	0.294
Difficulty getting up	0.378	0.265	1.459	0.86–2.45	0.155
Triggers					0.759
Hunger	−0.014	0.443	0.986	0.41–2.34	0.974
Intense physical activities	−0.172	0.556	0.842	0.28–2.50	0.757
Climate change	0.664	0.532	1.942	0.68–5.50	0.212
Menstruation	−0.119	0.478	0.888	0.34–2.26	0.803
Stress	0.264	0.434	1.301	0.55–3.04	0.543
Lack of sleep	0.065	0.504	1.067	0.39–2.86	0.897

SE, standard error; OR, odds ratio; VAS, Visual Analog Scale

### 3.5 Network Analysis Findings

**FIGURE 1:**
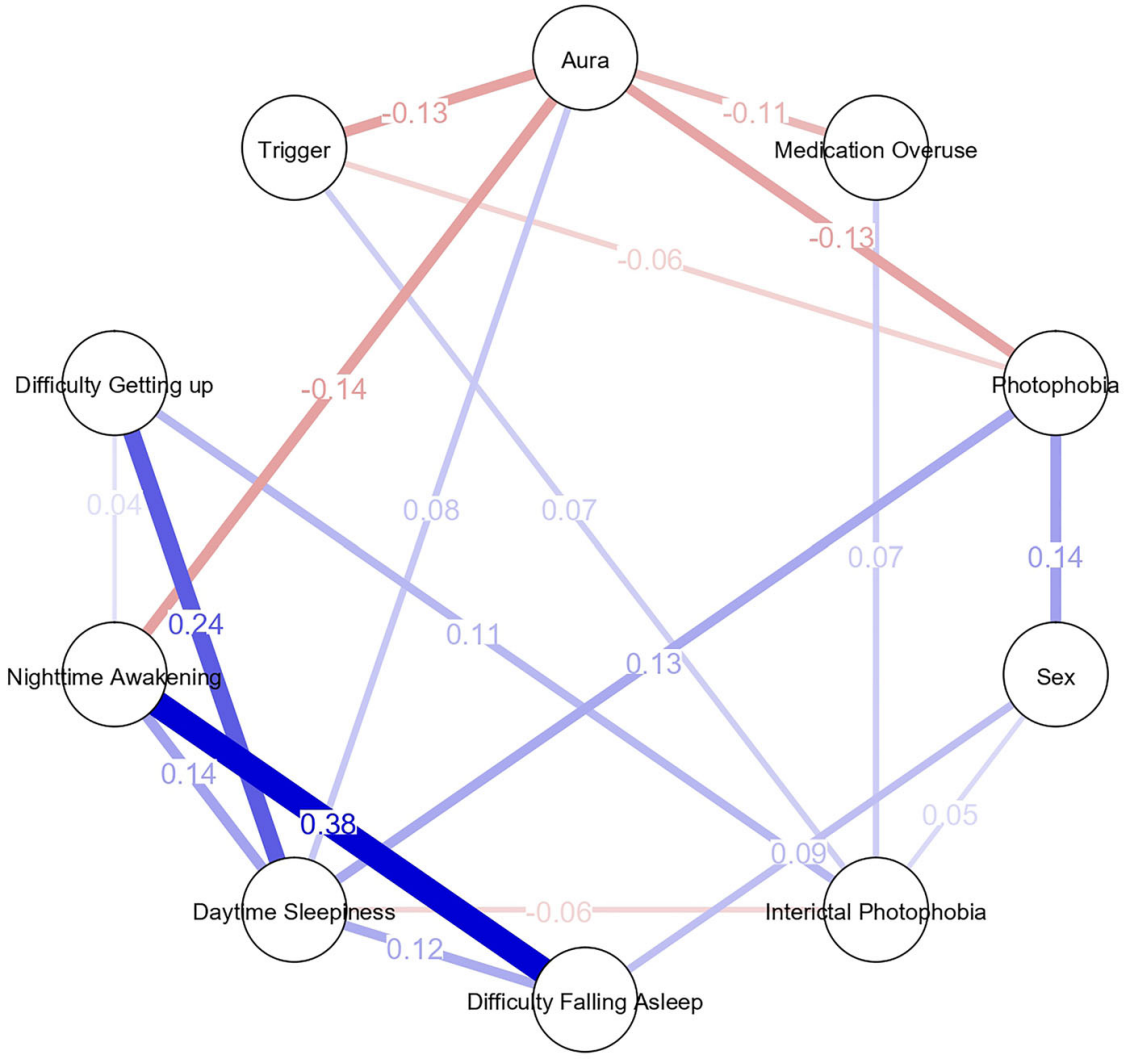
Network analysis of relationships between photophobia, sleep disturbances, and other migraine‐related symptoms in patients with EM.

**FIGURE 2:**
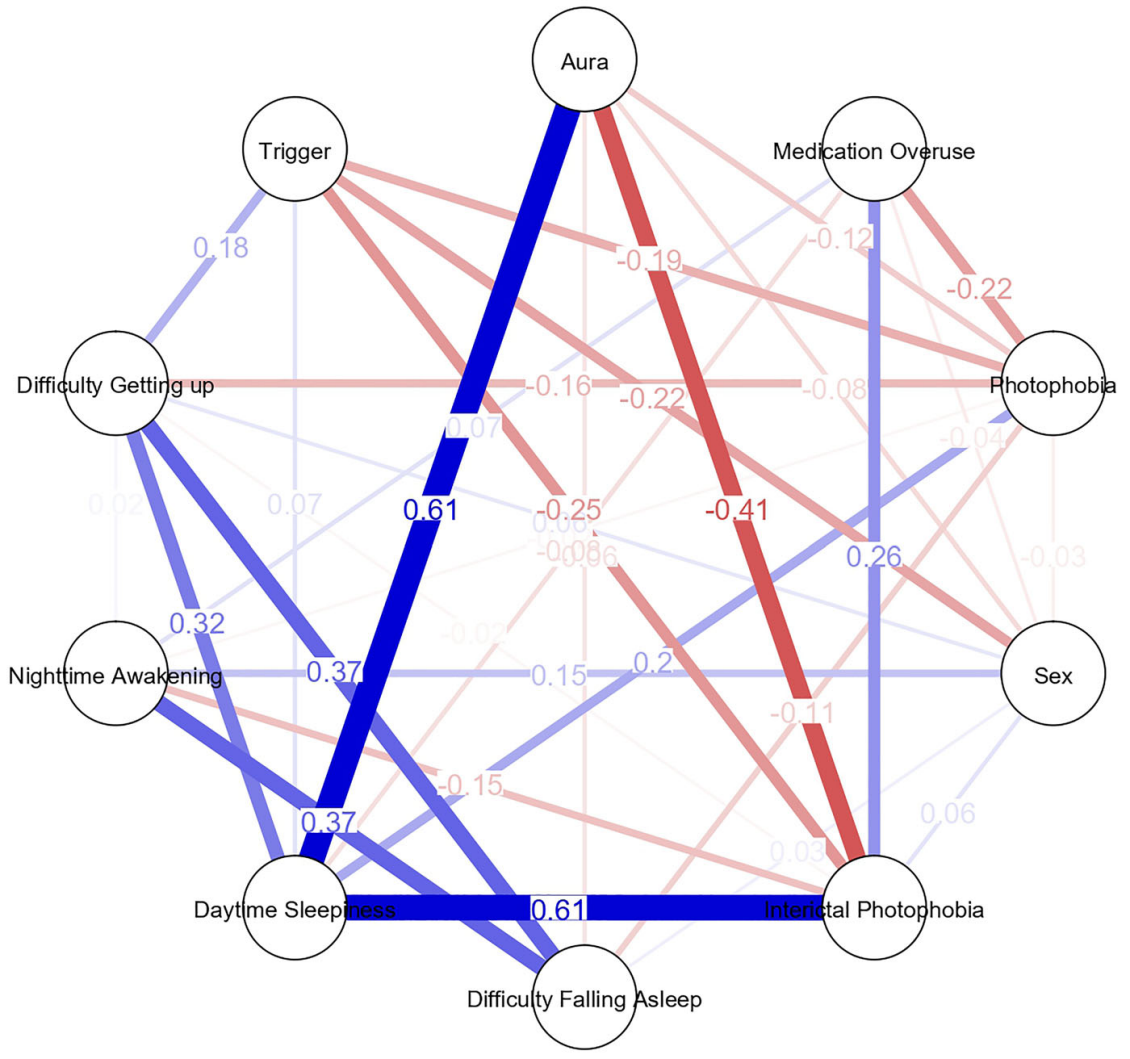
Network analysis of relationships between photophobia, sleep disturbances, and other migraine‐related symptoms in patients with CM.

Network analysis was performed to explore the relationships between photophobia, sleep disturbances, and other migraine‐related symptoms. In CM patients, interictal photophobia demonstrated strong associations with medication overuse and daytime sleepiness. These relationships were visualized in the network graph, where the thicker blue edges indicated significant positive correlations. In contrast, the connections between photophobia and other symptoms in EM patients were limited, with weaker associations visualized as thinner edges (Figures 1 and 2). Additionally, the network analysis revealed that aura was less strongly associated with interictal photophobia and other symptoms in CM patients than in EM patients. These findings underscore the central role of interictal photophobia in CM, where it appears to interact more robustly with other clinical factors.

Network comparison revealed that, after adjusting for age, sex, and MOH, the overall structure was denser in CM than in EM, with higher centrality around pain‐ and photophobia‐related nodes (Figures S1 and S3). In contrast, two weak negative correlations between Aura–Interictal photophobia and Aura–Trigger emerged only in EM, and bootstrap analyses showed wide 95% confidence intervals that crossed zero, indicating statistical fragility of these associations (Figure S2).

### 3.6 Key Comparisons Between Episodic and Chronic Migraine

1. **Medication overuse**: CM patients had a significantly higher prevalence of medication overuse than EM patients (57.1% vs. 19.6%, *p* < 0.001).
2. **Interictal photophobia**: The severity of interictal photophobia was similar in both groups, with most patients reporting severe forms (*p* = 0.498).
3. **Sleep disturbances**: Difficulty falling asleep showed a trend toward being more frequent in CM patients, though it was not statistically significant (*p* = 0.051).
4. **Aura**: Aura was significantly more common in EM patients than CM patients (11.6% vs. 2.1%, *p* < 0.001).

## 4 Discussion

This study aimed to examine the relationship between ictal and interictal photophobia, sleep disturbances, and the chronicity of migraine. We found ictal photophobia was present in 89.8% of the migraine patients, and interictal photophobia was present in 93.4% of the patients. 61.0% of the patients with interictal photophobia reported severe intensity. 44.9% of patients had difficulty falling asleep, 33.6% had daytime sleepiness, 43.1% had nighttime awakening, 12.7% had experienced nightmares, and 44.6% had trouble getting up. Our results showed no significant differences between EM and CM patients concerning ictal photophobia, interictal photophobia, and sleep disturbances. In the logistic regression model, medication overuse and aura were significant and independent predictors for CM. In network analysis, in individuals with CM, there was a stronger and more pronounced relationship with symptoms such as interictal photophobia, daytime sleepiness, and aura. These findings suggest that interictal photophobia may be associated with network‐level characteristics related to migraine progression.

To further explore the interplay among these symptoms and assess the robustness of these associations, supplementary network analyses were conducted. As detailed in Figures S1–S3, the CM network demonstrated higher density and node centrality around pain and photophobia, supporting a more integrated symptom interaction pattern. Conversely, in EM, two weak negative connections (Aura–Interictal photophobia and Aura–Trigger) were observed but proved statistically fragile after bootstrap testing, suggesting sample‐dependent variability rather than actual inhibitory effects. Together, these findings indicate that the transition from episodic to chronic migraine may reflect a structural reorganization of symptom interconnections— where sensory and sleep‐related factors become more interconnected within the chronic state. Comments should be interpreted in conjunction with Figures S1–S3 and Table S1 to provide an integrated view of the network‐based findings.

Most migraine sufferers avoid light during attacks, but some exhibit light sensitivity even just before or in the absence of a headache (Leibovit‐Reiben et al. 2024). Photophobia is experienced by 80% to 90% of individuals during a migraine episode (McAdams et al. 2020). It is frequently reported as an aggravation of headache by light, abnormal sensitivity to light, and eye discomfort/pain (Noseda et al. 2019). In the Migraine Symptoms and Treatment (MAST) study in the United States, 49.1% of participants reported that photophobia was the most bothersome symptom (Munjal et al. 2020). Similar to the high frequency reported in the literature (Epskamp et al. 2012; Sharp et al. 2024), we found that ictal photophobia was present in 89.8% of migraine patients and interictal photophobia was present in 93.4% of them. The modestly higher prevalence of interictal photophobia may reflect a masking effect, where severe pain and nausea during attacks may overshadow awareness of light sensitivity. In contrast, during migraine‐free periods, patients may be more conscious of discomfort in daily activities. This finding might also be influenced by individual variability in symptom amplification during attacks.

Several pathophysiological mechanisms have been proposed to explain migraine‐related photophobia, including altered visual–trigeminal interactions, trigeminal nociceptor sensitization, visual cortical hypersensitivity, abnormal brainstem habituation, and autonomic dysregulation (Noseda and Burstein 2011). Increasing evidence suggests that these mechanisms are not mutually exclusive but rather represent interconnected components of a broader multisensory network dysfunction underlying photophobia in migraine (Goadsby et al. 2017).

Photophobia in migraine appears to reflect abnormal integration of visual and nociceptive information within central sensory processing networks rather than a purely peripheral ocular phenomenon. Neuroimaging studies have demonstrated exaggerated activation of visual cortical regions, particularly the cuneus and lingual gyrus, in response to light stimulation in migraine patients during both ictal and interictal periods, indicating persistent cortical hyperresponsiveness (Boulloche et al. 2010; Denuelle et al. 2011). In parallel, nociceptive input arising from cranial vascular structures is transmitted through trigeminovascular pathways to posterior thalamic nuclei and subsequently relayed to higher cortical regions. The convergence of trigeminovascular and photic signals at thalamocortical and cortical levels provides a neuroanatomical substrate for multisensory hypersensitivity, including photophobia (Goadsby et al. 2017).

Importantly, the persistence of visual cortical activation even after headache resolution suggests that photophobia is not solely dependent on acute pain but rather reflects a sustained state of altered sensory processing. Subcortical structures, including the hypothalamus and brainstem, may further modulate this process by influencing both circadian regulation and pain‐related signaling, thereby amplifying sensory hypersensitivity during both ictal and interictal periods. Together, these mechanisms support a cyclic model of sensory network dysfunction in migraine, in which repeated activation of trigeminothalamic and visual pathways promotes maladaptive neuroplastic changes, contributing to the maintenance of interictal photophobia and potentially contributing to migraine progression (Goadsby et al. 2017).

From a network perspective, the relatively high centrality of interictal photophobia observed in CM patients suggests that this symptom occupies an important position within symptom interactions, particularly in relation to sleep‐related nodes, and may reflect stronger coupling between sensory hypersensitivity and broader migraine‐related network features.

Individuals with migraine who experience photophobia are at a higher risk of developing chronic migraine (Ashina et al. 2021). Over 80% of patients with chronic migraine reported experiencing severe photophobia (Diel et al. 2018). In line with this high overall burden, our study further shows that photophobia remains prominent beyond headache attacks, with 58.7% of CM patients reporting severe interictal intensity. In addition to sensory hypersensitivity, sleep disturbances represent another core clinical dimension in migraine. Patients with migraine frequently report poor sleep quality and disturbances, which serve both as triggers for migraine attacks and as consequences of these attacks. Common manifestations include difficulty falling asleep, trouble maintaining sleep, self‐reported reduced sleep duration, excessive daytime sleepiness, and not feeling refreshed after sleep. Despite significant progress in research over the past two decades, the exact nature and direction of the relationship between sleep and migraine remain unclear (Munjal et al. 2020).

Beyond the independent effects of sleep disturbance, the interaction between photophobia and sleep represents a particularly important pathway in migraine. There is considerable evidence suggesting a potential connection between photophobia and sleep in migraine. Clinically, two open‐label studies have shown that green light alleviates headaches, reduces photophobia, and improves sleep (Lipton et al. 2023; Martin et al. 2021). According to data from the American Registry for Migraine Research (ARMR), migraine patients with photophobia reported significantly worse sleep quality compared to those without photophobia, and increased levels of photophobia were linked to poorer sleep‐related outcomes (Munjal et al. 2020). The authors suggested that photophobia may act as a risk factor for sleep disorders in individuals with migraines. In our study, the differences in the frequency of photophobia and sleep disturbances between chronic and episodic migraine were relatively modest. However, network analysis revealed that chronic migraine is distinguished by stronger interconnections and greater centrality of these symptoms within the clinical network. These findings suggest a shift from isolated symptom occurrences toward a more integrated pattern of symptom interactions in chronic migraine.

From a mechanistic perspective, the circadian system may connect photophobia and light exposure. Light, particularly bright light, is the most potent zeitgeber, synchronizing the internal biological clock with external environmental cues while the circadian system regulates the sleep–wake cycle (Golombek and Rosenstein 2010). Although patients with photophobia often avoid bright or glaring light to minimize discomfort, it is unlikely that they avoid daytime light exposure entirely. Instead, such behavioral light‐avoidance patterns may reduce exposure to natural daylight and increase reliance on artificial indoor lighting, particularly blue light–emitting screens during evening hours. This imbalance—insufficient daytime bright light and excessive nighttime blue light exposure—may lead to circadian misalignment and contribute to the sleep disturbances frequently observed in migraine (Finan et al. 2013; Sharp et al. 2024; Vgontzas and Pavlović 2018).

Intrinsically photosensitive retinal ganglion cells (ipRGCs), which respond to bright light, project to the suprachiasmatic and Edinger–Westphal nuclei involved in circadian regulation and pupillary responses, as well as to thalamic pain‐processing pathways (Finan et al. 2013; Golombek and Rosenstein 2010). Beyond their role in circadian entrainment, recent experimental data indicate that blue light stimulation within the melanopsin‐sensitive spectrum may preferentially activate ipRGCs and lower the threshold for cortical spreading depression (Nagata et al. 2024). Moreover, enhanced pupillary responses consistent with increased ipRGC sensitivity have been reported in patients with migraine compared to controls (Nagata et al. 2024). Together, these findings suggest that ipRGCs may contribute to the pathophysiological relationship between photophobia and migraine (Finan et al. 2013; Golombek and Rosenstein 2010; Nagata et al. 2024). Future studies may further elucidate the role of daytime and nocturnal blue light exposure in migraine.

In addition to circadian misalignment, sleep disturbance itself may further exacerbate migraine‐related network dysregulation by increasing pain sensitivity and promoting central sensitization. Experimental and clinical studies suggest that sleep deprivation alters hypothalamic and brainstem function and weakens endogenous pain modulation, thereby lowering pain thresholds and potentially facilitating stronger coupling between sensory and pain‐processing networks in migraine (Alstadhaug 2009; Finan et al. 2013).

1Schiano di Cola found that more than 60% of patients reported a notable improvement in ictal photophobia during treatment with Galcanezumab (Ceccardi et al. 2023). The findings of this study may highlight the need for a multifaceted approach to CM management that considers both photophobia and sleep disturbances. Treatment strategies should focus not only on acute migraine relief but also on managing persistent symptoms that may contribute to the chronicity of the disorder. Interventions targeting photophobia, such as light therapy or the use of photophobia‐specific treatments, could be beneficial for CM patients. Moreover, pharmacological treatments that address central sensitization may help alleviate both photophobia and other migraine symptoms. Additionally, improving sleep quality through cognitive behavioral therapy or pharmacological interventions could reduce the severity of sleep disturbances and migraine frequency, ultimately improving patients’ quality of life.

### 4.1 Limitations

This study has several limitations. Although the present study demonstrates strong associations between photosensitivity, sleep disturbances, and migraine chronicity, the retrospective design precludes conclusions about causality. It is plausible that chronic migraine itself, through mechanisms such as sustained central sensitization and altered hypothalamic regulation, could further amplify light sensitivity and disrupt sleep. Alternatively, persistent photophobia and poor sleep quality may contribute to central sensitization and progression toward chronic migraine. This bidirectional interaction warrants further investigation in longitudinal and interventional studies to better clarify the temporal and mechanistic pathways involved. Second, both photophobia and sleep disturbances were assessed based on self‐reports rather than validated scales, which may introduce reporting bias. Future studies should utilize structured questionnaires to provide more robust data. Third, the single‐region sample may limit the generalizability of our findings. Although the results provide valuable insights, more extensive multicenter studies with more diverse populations must confirm the observed associations. Finally, while network analysis offers a novel perspective, it is primarily exploratory and should be interpreted with traditional statistical methods.

## 5 Conclusion

In conclusion, we found that 89.8% of the migraine patients had ictal photophobia, and 93.4% of the patients had interictal photophobia. While no significant differences were found between episodic and chronic migraine patients in terms of ictal photophobia, interictal photophobia, and sleep disturbances, network analysis revealed that in CM patients, there was a stronger and more pronounced relationship with symptoms such as interictal photophobia, daytime sleepiness, and aura. By addressing the underlying mechanisms of photophobia and sleep disturbances and implementing targeted interventions, the chronicity of migraine may be better managed. Future research should focus on validating these findings and exploring integrated therapeutic approaches that address both primary and secondary symptoms.

## Author Contributions

Conceptualization: Seden Demirci, Aynur Özge, Derya Uludüz. Methodology: Seden Demirci, Aynur Özge, Derya Uludüz, Software: Fatma Yardibi, Damla Hazal Sucu, Aynur Özge. Data curation: Seden Demirci, Ezgi Uludüz, Sevim Eyüboğlu, Aynur Özge, Derya Uludüz. Investigation: Seden Demirci, Ezgi Uludüz, Sevim Eyüboğlu, Utku Topbaş, Derya Uludüz. Validation: Fatma Yardibi, Damla Hazal Sucu, Aynur Özge, Derya Uludüz. Formal analysis: Fatma Yardibi, Damla Hazal Sucu, Aynur Özge, Derya Uludüz. Supervision: Seden Demirci, Utku Topbaş, Aynur Özge, Derya Uludüz. Funding acquisition: Derya Uludüz. Visualization: Fatma Yardibi, Damla Hazal Sucu, Aynur Özge. Project administration: Seden Demirci, Aynur Özge, Derya Uludüz. Resources: Seden Demirci, Sevim Eyüboğlu, Aynur Özge, Derya Uludüz. Writing – original draft: Seden Demirci, Aynur Özge. Writing – review and editing: Seden Demirci, Utku Topbaş, Aynur Özge, Derya Uludüz.

## Funding

The authors have nothing to report.

## Ethics Statement

The study received approval from the Ethics Committee of Akdeniz University Faculty of Medicine (Approval Date: April 25, 2024, Decision No: 254). Written informed consent was obtained from participants.

## Conflicts of Interest

The authors declare that they have no known competing financial interests or personal relationships that could have appeared to influence the work reported in this paper.

## Supporting information




**Supporting Information**: brb371311‐sup‐0001‐SuppMat.docx

## Data Availability

Upon reasonable request, the corresponding author will provide access to the datasets generated and/or analyzed during this study.

## Footnotes

^1^
In the text, “Schiano di Cola” should be corrected to “Schiano di Cola et al.”. In the text, the citation “(Ceccardi et al., 2023)” should be corrected to “(Schiano di Cola et al., 2023)”.

## References

[brb371311-bib-0024] Alstadhaug, K. B. 2009. “Migraine and the Hypothalamus.” Cephalalgia 29, no. 8: 809–817. 10.1111/j.1468-2982.2008.01814.x. 19604254

[brb371311-bib-0017] Ashina, S. , A. Lyngberg , and R. Jensen . 2021. “Headache Characteristics and Chronification of Migraine and Tension‐Type Headache: A Population‐Based Study.” Cephalalgia 30, no. 8: 943–954. 10.1177/0333102409357958. 20656705

[brb371311-bib-0016] Boulloche, N. , M. Denuelle , P. Payoux , N. Fabre , Y. Trotter , and G. Geraud . 2010. “Photophobia in Migraine: An Interictal PET Study of Cortical Hyperexcitability and Its Modulation by Pain.” Journal of Neurology, Neurosurgery, and Psychiatry 81: 978–984. 10.1136/jnnp.2009.190223. 20595138

[brb371311-bib-0015] Denuelle, M. , N. Boulloche , P. Payoux , N. Fabre , Y. Trotter , and G. Géraud . 2011. “A PET Study of Photophobia During Spontaneous Migraine Attacks.” Neurology 76: 213–218. 10.1212/WNL.0b013e3182074a57. 21148120

[brb371311-bib-0018] Diel, R. J. , Z. A. Kroeger , R. C. Levitt , et al. 2018. “Botulinum Toxin A for the Treatment of Photophobia and Dry Eye.” Ophthalmology 125, no. 1: 139–140. 10.1016/j.ophtha.2017.09.031. 29110944 PMC5741464

[brb371311-bib-0009] Epskamp, S. , A. O. J. Cramer , L. J. Waldorp , V. D. Schmittmann , and D. Borsboom . 2012. “Network Visualizations of Relationships in Psychometric Data.” Journal of Statistical Software 48, no. 4: 1–18. 10.18637/jss.v048.i04.

[brb371311-bib-0022] Finan, P. H. , B. R. Goodin , and M. T. Smith . 2013. “The Association of Sleep and Pain: An Update and a Path Forward.” The Journal of Pain 14, no. 12: 1539–1552. 10.1016/j.jpain.2013.08.007. 24290442 PMC4046588

[brb371311-bib-0001] GBD 2016 Headache Collaborators . 2018. “Global, Regional, and National Burden of Migraine and Tension‐Type Headache, 1990–2016: a Systematic Analysis for the Global Burden of Disease Study 2016.” Lancet Neurology 17, no. 11: 954–976. 10.1016/S1474-4422(18)30322-3. 30353868 PMC6191530

[brb371311-bib-0014] Goadsby, P. J. , P. R. Holland , M. Martins‐Oliveira , J. Hoffmann , C. Schankin , and S. Akerman . 2017. “Pathophysiology of Migraine: A Disorder of Sensory Processing.” Physiological Reviews 97, no. 2: 553–622. 10.1152/physrev.00034.2015. 28179394 PMC5539409

[brb371311-bib-0021] Golombek, D. A. , and R. E. Rosenstein . 2010. “Physiology of Circadian Entrainment.” Physiological Reviews 90: 1063–1102. 20664079 10.1152/physrev.00009.2009

[brb371311-bib-0008] Headache Classification Committee of the International Headache Society (IHS) the International Classification of Headache Disorders . 2018. 3rd edition. “Cephalalgia” 38: 1–211. 10.1177/0333102417738202. 29368949

[brb371311-bib-0004] Leibovit‐Reiben, Z. , G. Dumkrieger , D. W. Dodick , et al. 2024. “Photophobia Contributes to Migraine‐Associated Disability and Reduced Work Productivity: Results from the American Registry for Migraine Research (ARMR).” Journal of Neuro‐Ophthalmology 44, no. 2: 259–266. 10.1097/WNO.0000000000001967. 37581595

[brb371311-bib-0020] Lipton, R. B. , A. Melo‐Carrillo , M. Severs , et al. 2023. “Narrow Band Green Light Effects on Headache, Photophobia, Sleep, and Anxiety Among Migraine Patients: An Open‐Label Study Conducted Online Using Daily Headache diary.” Frontiers in Neurology 14: 1282236. 10.3389/fneur.2023.1282236. 37859647 PMC10582938

[brb371311-bib-0002] Malone, C. , A. Wachholtz , and A. Bhowmick . 2015. “Migraine: Treatments, Comorbidities, and Quality of Life, in the USA.” Journal of Pain Research 8: 537. 10.2147/JPR.S88207. 26316804 PMC4540217

[brb371311-bib-0019] Martin, L. F. , A. M. Patwardhan , S. V. Jain , et al. 2021. “Evaluation of Green Light Exposure on Headache Frequency and Quality of Life in Migraine Patients: A Preliminary One‐Way Cross‐Over Clinical Trial.” Cephalalgia 41, no. 2: 135–147. 10.1177/0333102420956711. 32903062 PMC8034831

[brb371311-bib-0010] Mcadams, H. , E. A. Kaiser , A. Igdalova , et al. 2020. “Selective Amplification of ipRGC Signals Accounts for Interictal Photophobia in Migraine.” Proceedings of the National Academy of Sciences of the United States of America 117, no. 29: 17320–17329. 10.1073/pnas.2007402117. 32632006 PMC7382295

[brb371311-bib-0003] Mulleners, W. M. , S. K. Aurora , E. P. Chronicle , R. Stewart , S. Gopal , and P. J. Koehler . 2001. “Self‐reported Photophobic Symptoms in Migraineurs and Controls Are Reliable and Predict Diagnostic Category Accurately.” Headache 41, no. 1: 31–39. 10.1046/j.1526-4610.2001.111006031.x. 11168601

[brb371311-bib-0012] Munjal, S. , P. Singh , M. L. Reed , et al. 2020. “Most Bothersome Symptom in Persons With Migraine: Results From the Migraine in America Symptoms and Treatment (MAST) Study.” Headache 60: 416–429. 10.1111/head.13708. 31837007 PMC7027490

[brb371311-bib-0023] Nagata, E. , M. Takao , H. Toriumi , et al. 2024. “Hypersensitivity of Intrinsically Photosensitive Retinal Ganglion Cells in Migraine Induces Cortical Spreading Depression.” International Journal of Molecular Sciences 25, no. 14:7980. 10.3390/ijms25147980. 39063222 PMC11276861

[brb371311-bib-0011] Noseda, R. , D. Copenhagen , and R. Burstein . 2019. “Current Understanding of Photophobia, Visual Networks and Headaches.” Cephalalgia 39, no. 13: 1623–1634. 10.1177/0333102418784750. 29940781 PMC6461529

[brb371311-bib-0013] Noseda, R. , and R. Burstein . 2011. “Advances in Understanding the Mechanisms of Migraine‐Type Photophobia.” Current Opinion in Neurology 24, no. 3: 197–202. 10.1097/WCO.0b013e3283466c8e. 21467933 PMC4502959

[brb371311-bib-0005] Pinheiro, C. F. , J. R. Moreira , G. F. Carvalho , L. Zorzin , F. Dach , and D. Bevilaqua‐Grossi . 2021. “Interictal Photophobia and Phonophobia Are Related to the Presence of Aura and High Frequency of Attacks in Patients With Migraine.” Applied Sciences 11, no. 6:2474. 10.3390/app11062474.

[brb371311-bib-0025] Schiano di Cola, F. , G. Ceccardi , M. Bolchini , S. Caratozzolo , et al. 2023. “Photophobia and Migraine Outcome During Treatment With Galcanezumab.” Frontiers in Neurology 13: 1088036. 36742057 10.3389/fneur.2022.1088036 PMC9889984

[brb371311-bib-0007] Sharp, N. , M. J. Burish , K. B. Digre , et al. 2024. “Photophobia Is Associated With Lower Sleep Quality in Individuals With Migraine: Results From the American Registry for Migraine Research (ARMR).” The Journal of Headache and Pain 25, no. 1: 55. 10.1186/s10194-024-01756-9. 38609895 PMC11015590

[brb371311-bib-0006] Vgontzas, A. , and J. M. Pavlović . 2018. “Sleep Disorders and Migraine: Review of Literature and Potential Pathophysiology Mechanisms.” Headache 58: 1030–1039. 10.1111/head.13358. 30091160 PMC6527324

