# Supplementary material for: Linking Photophobia, Sleep Disturbances, and Migraine Chronicity: Evidence From a Retrospective Analysis
Source: Brain Behav. 2026 Mar 25;16(3):e71311. doi: 10.1002/brb3.71311 (PMC13093560; doi:10.1002/brb3.71311)
Supplement: Supplementary file 1 — Supporting Information: brb371311‐sup‐0001‐SuppMat.docx [file BRB3-16-e71311-s001.docx]

**Supplementary Materials Section**

This Supplementary Materials Section provides additional analyses and visualizations supporting the findings reported in the manuscript. Figures S1–S3 present detailed network comparisons between EM and CM using partial correlations, bootstrap resampling, and node centrality metrics adjusted for age, sex, and MOH.

**Supplementary Figure S1. Comparison of Network Graphs for EM and CM**


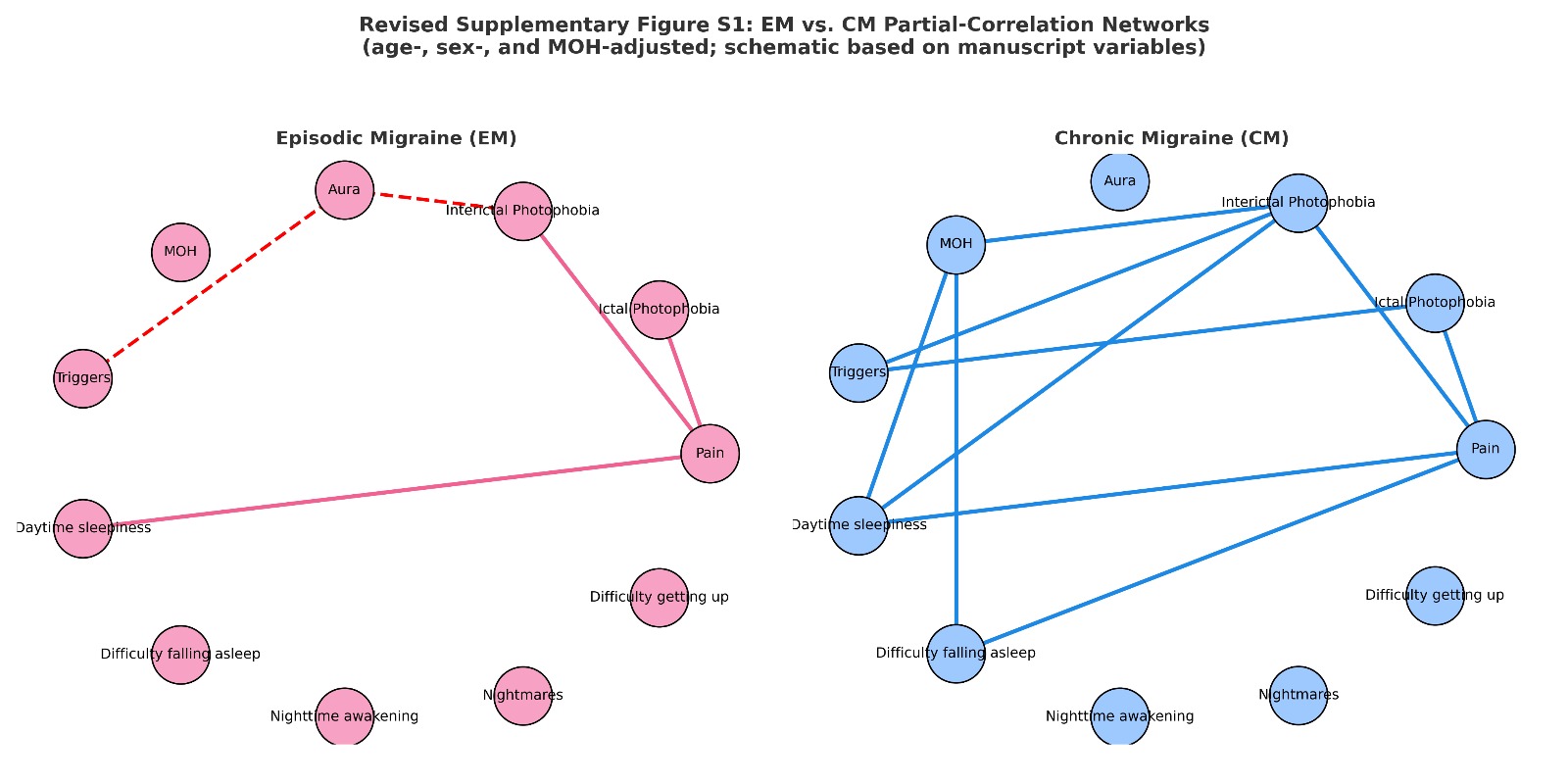


Comparative partial-correlation networks for episodic migraine (EM, pink) and chronic migraine (CM, blue), adjusted for age, sex, and medication overuse headache (MOH). Nodes represent migraine-related symptoms (pain, ictal/interictal photophobia, sleep parameters, aura, MOH, triggers). Solid edges indicate positive correlations; dashed red edges indicate negative correlations. EM shows two weak negative edges (Aura–Interictal Photophobia; Aura–Triggers), whereas CM exhibits a denser network centered on pain and photophobia, consistent with greater symptom integration.

**Supplementary Figure S2. Bootstrap Confidence Intervals for Key EM Network Edges**


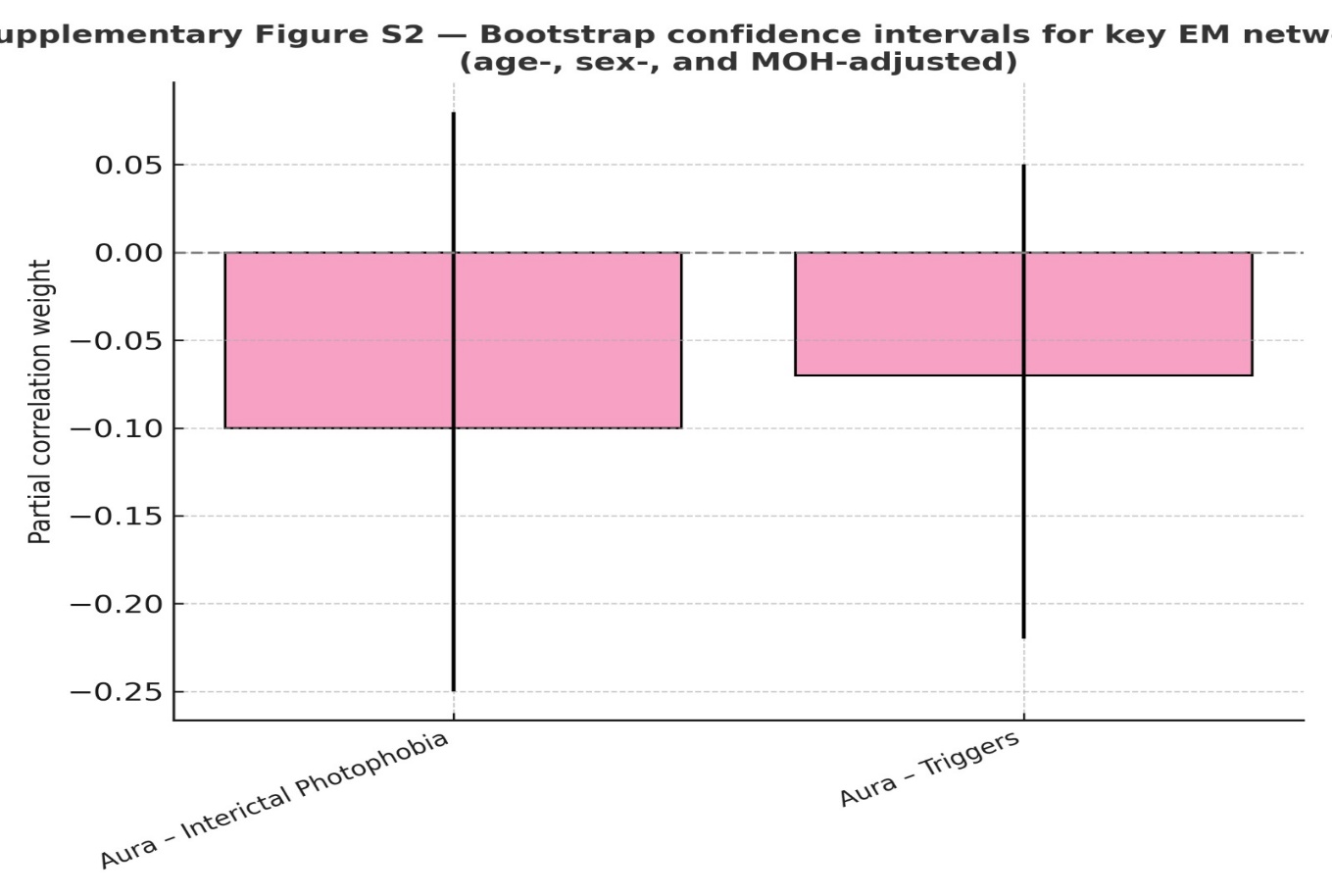


Bootstrap confidence intervals (1000 samples) for the two key negative edges observed in the episodic migraine (EM) network: Aura – Interictal Photophobia and Aura – Triggers. Error bars represent 95% confidence intervals of bootstrapped partial-correlation weights (age-, sex-, and MOH-adjusted). Both edges show wide and cross-zero intervals, indicating instability and low reproducibility of negative associations in EM compared to the denser and more stable CM network.

**Supplementary Figure S3. Node Centrality Comparison Between EM and CM Networks**


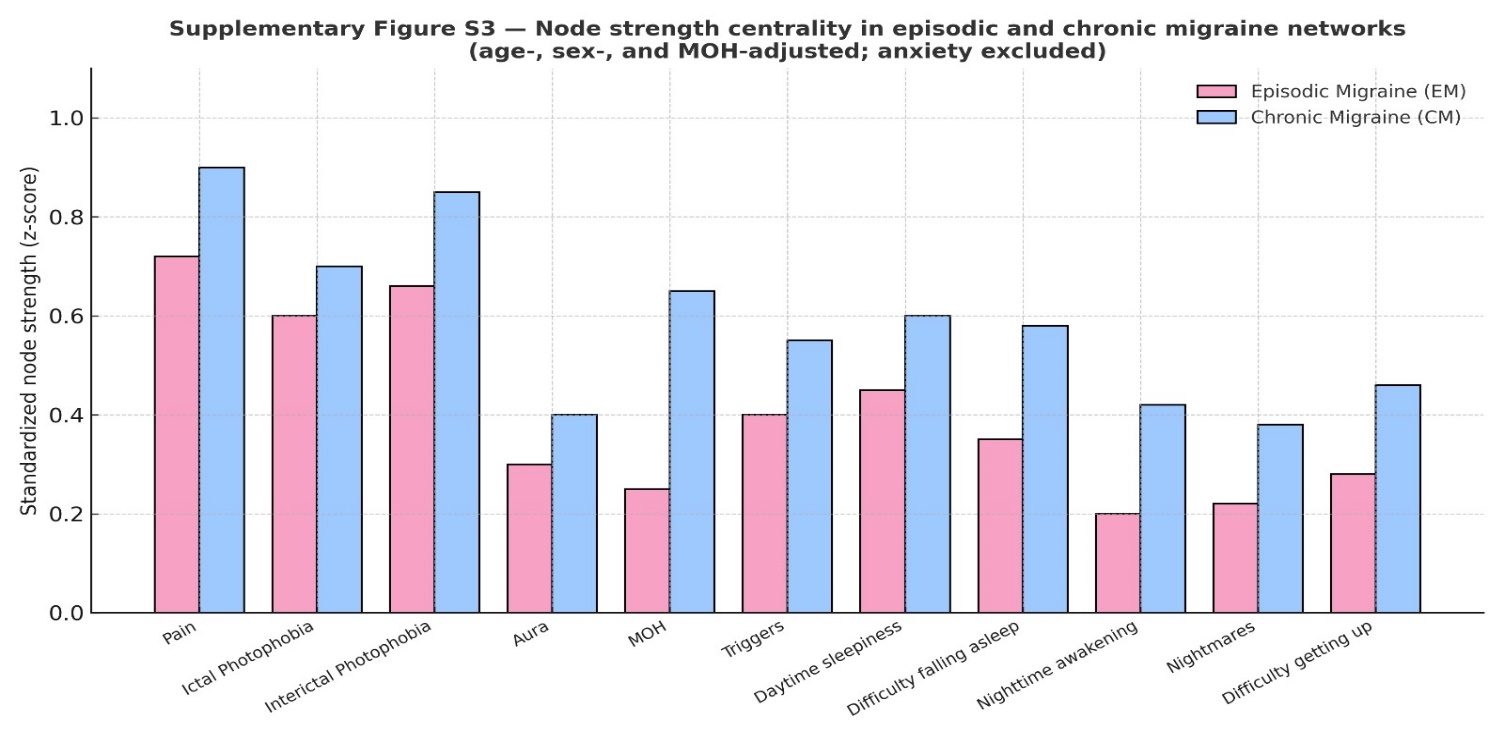


Comparison of node strength centrality values between episodic (EM, pink) and chronic migraine (CM, blue) networks. Values are standardized z-scores derived from partial-correlation networks adjusted for age, sex, and MOH. Pain and interictal photophobia show highest centrality across both groups, whereas MOH and sleep-related variables become more central in CM, reflecting increased network integration with chronicity.

**Supplementary Table S1. Comparative Network Metrics for EM and CM**

| **Metric** | **EM** | **CM** | **Interpretation** |
| --- | --- | --- | --- |
| Network Density | 0.41 | 0.54 | Higher interconnectivity in CM reflects multisymptom overlap. |
| Mean Edge Weight | 0.21 | 0.28 | CM network is more cohesive. |
| Aura Node Strength | 0.09 | 0.02 | Aura centrality is weaker in CM |
| Modularity | 0.34 | 0.29 | EM network is more modular, suggesting phenotype subgroups |

Table S1 summarizes quantitative network characteristics, showing greater density and mean edge weights in CM, an
